# Effects of strength training and detraining considering maturity status in youth highly trained basketball players

**DOI:** 10.1371/journal.pone.0317879

**Published:** 2025-02-12

**Authors:** Oliver Gonzalo-Skok, Jorge Arede, Thomas Dos’Santos

**Affiliations:** 1 Department of Communication and Education, Universidad Loyola Andalucía, Seville, Spain; 2 School of Education, Polytechnic Institute of Viseu, Viseu, Portugal; 3 Department of Sports Sciences, Exercise and Health, University of Trás-os-Montes and Alto Douro, Vila Real, Portugal; 4 Research Center in Sports Sciences, Health Sciences and Human Development, CIDESD, University of Trás-os-Montes and Alto Douro, Vila Real, Portugal; 5 Department of Sport and Exercise Sciences, Manchester Institute of Sport, Manchester Metropolitan University, Manchester, United Kingdom; Università degli Studi di Milano: Universita degli Studi di Milano, ITALY

## Abstract

The study analyzed whether maturation affects young basketball players’ strength training and detraining adaptations. Thirty-five youth male basketballers (U-13 to U-15) performed eight weeks of strength training twice weekly, followed by eight weeks without strength training, maintaining their basketball practices. Changes in performance were assessed in three maturity groups based on years from/to age of peak height velocity (PHV): pre-PHV (-1.51 ±  0.62, n  =  9), mid-PHV (0.11 ±  0.45, n  =  17), and post-PHV (1.31 ±  0.42, n  =  9). They were tested on bilateral-vertical countermovement jump, unilateral vertical and horizontal jumping, unilateral triple horizontal jumping (3HJ), sprinting (25-m), and change of direction (COD) ability over single and multiple angles. All groups significantly (p  <  0.05; ES  =  0.42–1.10) improved unilateral horizontal jumping ability between pre-and post-test. Unilateral vertical jumping significantly improved in mid-PHV and post-PHV between pre- and post- (p  <  0.05; ES  =  0.46–0.61) and pre- to detraining (p  <  0.05; ES  =  0.51–1.01). Pre-PHV and mid-PHV significantly enhanced 3HJ between pre- and post- (p  <  0.05; ES  =  0.72–1.15) and de-training (p  <  0.05; ES  =  0.61–1.11). COD ability significantly improved in mid-PHV between pre- and post- and detraining (p  <  0.05; ES  =  0.47–0.80). Left horizontal jumping at post-test and detraining was significantly (p  <  0.05; ES  =  1.56 – 1.73) greater in post-PHV and mid-PHV than pre-PHV. The combined training had favorable outcomes for most unilateral jumps, particularly those with a horizontally oriented force vector, independent of maturity status. Moreover, the subsequent detraining period positively affected specific high-intensity actions, albeit variations were noted across different maturation stages. These findings can help practitioners to design effective strength training programmes during competitive basketball season for youth male basketballers.

## Introduction

Most basketball actions are performed unilaterally, variable, and multidirectional [1], such as jumping or cutting. Specifically, such actions performed in matches are unilateral and bilateral jumps, linear and curvilinear sprints, running in a zig-zag pattern, side-stepping, crossover cutting, or running back and forth repeatedly [2–4]. Youth basketball players from finalist teams demonstrate superior linear speed and multidirectional abilities than players from low-ranked teams [5]. In addition, those U-16 selected players to compete in an international basketball Championship also display better jumping and sprinting abilities [6]. Furthermore, elite and young male basketball players have been shown to change movement types every 1-3 seconds during a game [7,8]. Specifically, 97% of turns performed are between 0-180º (<45º =  54.6%; 90º =  27.2%; 135º =  7.4%; 180º =  8.3%; > 180º =  2.5%) being the most usual cutting angle during match play about 45º [9]. In this regard, it seems that unilateral and bilateral jumping in several directions and speed abilities (i.e., linear and change of direction (COD) speeds) are essential within decisive situations in basketball, and, thus, they should be included in testing batteries and training protocols.

An optimal training stimulus is critical for effective planning and programming during athlete development. In this regard, it is important to note that the optimal stimulus when training youth athletes should account for the maturity status, which is commonly registered through the age at peak height velocity (PHV) [10]. Developments in neural properties are responsible for the prepubertal training adaptations through increased intramuscular and intermuscular coordination, while strength and hypertrophy gains mainly occur post-PHV [10–12]. Despite such information being essential to developing optimal training programs in youth athletes, further insight is required regarding the effects and optimization of training during growth and maturation.

Sprinting and jumping abilities are fundamental locomotive skills that form part of the athletic motor skill spectrum [12,13]. Several training methods, such as resistance, plyometric, rotational flywheel, and combined training have been proposed to improve such skills in young basketball players [14–20]. It appears that pre-PHV and mid-PHV males may benefit more from plyometric training [13,21], whereas post-PHV may maximize gains in sprinting following either combined strength training [13,21] or strength training [22]. Although the previous data [13,21,22] show that maturation may play a role in the interaction effects between strength training and maturation, there is no information related to the impact on unilateral jumping (vertical and horizontal) and COD speed (single and multiple) in any population. Furthermore, no study has analyzed the effects of maturation on training adaptations in young basketball players, and thus, further information is warranted.

Strength training helps improve essential athletic skills and neuromuscular function [14–20]. In this regard, combined strength training and force-vector specific training are the most effective strategies for improving the above-mentioned abilities [1,14,17]. However, its cessation negatively impacts muscular strength [23] commonly known as detraining. During maturation, however, physiological changes (e.g., increased anabolic hormonal concentrations, central nervous system myelinization, etc.) may influence the decay in performance following the cessation of strength training. To our best knowledge, only one previous study examined the detraining effect of different maturity groups following the cessation of a strength training program in male students [22]. The authors found that the effects of detraining varied according to maturity status. Regarding strength and power, a greater loss was observed in the pre-PHV group, whereas the post-PHV group experienced a decline in sprint performance. All groups either maintained or improved their horizontal jump performance [22]. However, in youth basketball, no studies have investigated the detraining effect after the cessation of strength training on young of different maturity statuses maintaining the basketball training routine. As such, there is a need to understand how highly trained basketball players (i.e., 12 hours of practice per week and competing at the National and International levels) respond to force-vector-oriented training depending on their maturity status and the cessation effect as they continue playing basketball as it occurs before an important tournament. Thus, the main aims of the current study were: 1) to examine the effect of 8 weeks’ strength training on athletic performance in youth basketball athletes and establish whether the response differs between maturity status and 2) to investigate the impact of strength training cessation whilst maintaining basketball training over eight weeks on jumping, linear sprinting and COD speed in young highly trained basketball players. We hypothesized that both training response and detraining effects vary according to maturity status.

## Materials and methods

### Participants

Thirty-five young (U-13 to U-15), highly trained male basketball players (age: 13.3 ±  1.0 years; height: 171.9 ±  12.9 cm; body mass: 56.1 ±  11.6 kg, APHV, 0.00 ±  1.14 years) volunteered to participate in this study (recruitment period 28th September 2015 to 31st January 2016). The estimated total sample size for and effect size f of 0.25 for an ANOVA of repeated measures through within-factors at alpha of 0.05 according to G * power (version 3.1.9.6) was 30 participants (actual power =  0.839), but to accommodate possible dropouts, we enrolled 35 participants in this study. Data collection occurred during the second month (i.e., November) of the competitive season after a 2-month pre-season period. All players were training in a basketball club for at least three years and participated on average in approximately 12 hours of combined basketball (6-7 sessions), strength and plyometrics (2 sessions), speed, agility, and quickness (1 session) training and two competitive matches per week. At the time of the study, all players were competing at a national level (i.e., Spanish Basketball National League). Furthermore, some players (n =  9) were also competing at the international level (i.e., European and World Basketball Championships). This study was approved by the University of Zaragoza Institutional Review Board (Approval Number: PI14/00114) and conformed to the recommendations of the Declaration of Helsinki. Written informed consent was obtained from all participants prior to their inclusion in the study. For participants under the age of 18, consent was obtained from a parent or legal guardian.

### Design

Players were divided into three maturity groups for analysis based on the peak height velocity (PHV) offset: pre-PHV (n  =  9), mid-PHV (n  =  17), and post-PHV (n  =  9). Tests were performed on an indoor basketball court one week before strength training intervention (pre-test), one week after the training intervention (post-test), and eight weeks after the training period (detraining). After the initial strength training intervention period, all players continued their basketball practices (6-7 sessions) without any strength training until eight weeks later (i.e., detraining period). Tests included anthropometric measurements, bilateral and unilateral countermovement jump (CMJ) tests, a unilateral horizontal jump test, a triple unilateral horizontal jump test, a 25-m linear running sprint test (5-m, 10-m, and 20-m split times), a 10-m shuttle-sprint test (5 +  5 m) with one 180º change of direction (COD) performed both right (180º CODR) and left (180º CODL) legs, and a multiple COD test (V-cut test). Players were familiarized with the exercise procedures before the commencement (i.e., tests were within the battery testing carried out 5 times per season). They were asked not to perform intense exercise on the day before a test and to consume their last meal at least three hours before the scheduled test time.

### Procedures

#### Strength training intervention.

Participants performed two weekly additional training sessions (i.e., at least 48 h after the game and 48 h between training sessions) to their basketball training routine, for 8 consecutive weeks, with mean group adherence of 93% and a minimal individual requirement of 80% to be included in the study. All training sessions were supervised by two qualified S&C coaches (i.e., NSCA CSCS). Training consisted of two different strength training approaches. One training day primarily focused on vertical-oriented strength exercises (Table 1), while the 2nd day focused on horizontal-oriented strength exercises (Table 2). Each training session was divided into six main exercises organized from low to high-speed exercises performing both one compensatory and one complementary exercise between each set of each main exercise. Progressive overload was based on increasing volume, intensity, and/or complexity (Tables 1 and 2). Programming information is provided in Tables 1 and 2 and conforms with the resistance training recommendations for youth athletes (Lloyd, Cronin, et al., 2016b). A 2-minute resting period was provided between sets and exercises. Exercises were performed as fast as possible in the concentric phase and a controlled manner (i.e., ~  2 s) in the eccentric phase).

**Table 1:** Exercise progression for the vertical-oriented training.

Vertical-oriented training									
		Weeks 1-2		Weeks 3-4		Weeks 5-6		Weeks 7-8	
	Exercise	Intensity	Volume	Intensity	Volume	Intensity	Volume	Intensity	Volume
**Main**	Bilateral SQ	10% BM	2 S x 12 R	15% BM	2 S x 10 R	20% BM	2 S x 8 R	25% BM	2 S x 6 R
	Sumo SQ	10% BM	2 S x 12 R	15% BM	2 S x 10 R	20% BM	2 S x 8 R	25% BM	2 S x 6 R
	One-legged SQ	BM	2 S x 12 R	MB	2 S x 10 R	10% BM	2 S x 8 R	15% BM	2 S x 6 R
	Lunge	10% BM static	2 S x 12 R	10% BM dynamic	2 S x 12 R	20% BM static	2 S x 10 R	20% BM dynamic	2 S x 10 R
	Step-up from lunge	Low step	2 S x 8 R	High step	2 S x 8 R	MB low step	2 S x 8 R	MB high step	2 S x 8 R
	Unilateral vertical jump	BM	2 S x 5 R	BM	2 S x 6 R	MB	2 S x 5 R	MB	2 S x 6 R
**Compen/Compl**	Plank	Regular	25 s	Up & Down legs	25 s	Up & Down legs & keep 3 s	25 s	Up & Down + abd leg	25 s
	Side plank	Knee on the floor	15 s/side	Regular	15 s/side	Hip abduction	15 s/side	Elevated leg	15 s/side

*Note.* Compen/Compl: compensatory and complementary; SQ: squat, BM: body mass; low step: 30 cm step; MB: medicine ball; high step: 50 cm step. S: Sets; R: Reps; Abd: Abduction.

**Table 2:** Exercise progression for the horizontal-oriented training.

		Horizontal-oriented training							
		Weeks 1-2		Weeks 3-4		Weeks 5-6		Weeks 7-8	
	Exercise	Intensity	Volume	Intensity	Volume	Intensity	Volume	Intensity	Volume
**Main**	Hip thrust	BM (2 legs)	2 S x 12 R	10% BM (2 legs)	2 S x 12 R	BM (1 leg)	2 S x 10 R	10% BM (1 leg)	2 S x 10 R
	Hip extension	BM (2 legs)	2 S x 12 R	10% BM (2 legs)	2 S x 12 R	BM (1 leg)	2 S x 10 R	10% BM (1 leg)	2 S x 10 R
	Unilateral Deadlift	BM	2 S x 12 R	5% BM	2 S x 10 R	10% BM	2 S x 8 R	15% BM	2 S x 6 R
	Unilateral horizontal jump	BM	2 S x 5 R	2 jumps	2 S x 5 R	3 jumps	2 S x 5 R	4 jumps	2 S x 5 R
	Sprint 20-m	BM	3 R	5% BM	3 R	7% BM	3 R	10% BM	3 R
	COD	BM	3 R	5% BM	3 R	7% BM	3 R	10% BM	3 R
**Compen/Compl**	Plank	Regular	25 s	Up & Down legs	25 s	Up & Down legs & keep 3 s	25 s	Up & Down + abd leg	25 s
	Bird Dog	Regular	20 s/side	Dynamic	20 s/side	Up & Down legs & keep 3 s	20 s/side	Up & Down + abd leg	20 s/side

*Note.* Compen/Compl: compensatory and complementary; COD: change of direction, BM: body mass; S: Sets; R: Reps; Abd: Abduction.

#### Measurements.

Participants were familiarized with all tests. Before the jumping, speed, and COD testing, all players performed a typical pre-game warm-up, including low-intensity jogging (10 minutes), dynamic stretches (lunges, diver, lateral squat) (5 minutes), and moderate to high-intensity activities such as high-knees, butt kicks, cariocas, accelerations, decelerations, linear sprints, and changes of direction (5 minutes). Testing was performed in the following order: bilateral CMJ, unilateral countermovement jump, unilateral horizontal jump, triple unilateral horizontal jump, 25-m linear sprinting, 180° COD, and V-cut test. Players executed two warm-up trials (in each direction during COD tests) at 75% and 90% maximum effort before their maximum effort trials. The rest interval was 3 minutes between different tests.

#### Anthropometric measurements.

Each player was weighed (in kg) using a digital scale (*Seca Instruments Ltd., Hamburg, Germany*), and their stature was measured (in cm) with a stadiometer (*Holtain Ltd., Crymych, UK*). Maturity offset was predicted using a non-invasive method appropriate for the age range of the sample, considering anthropometric data (leg length and sitting height measured on a chair), and chronological age (Maturity offset = -9.236 +  0.0002708 x (Leg Length x Sitting Height) -0.001663 x (Age x Leg Length) +  0.007216 x (Age x Sitting Height) +  0.02292 x ((Body mass/Height) x 100) [24]. This measure was previously validated in a male longitudinal study in the range of 8 to 18 years old [25]. Age at peak height velocity (APHV) was calculated by subtracting maturity offset from the chronological age. Players were classified based on Mirwald classification where those showing APHV values < -1.00 were pre-PHV, -1.00 to 1.00 were mid-PHV, and > 1.00 were post-PHV [24]. All measurements were recorded by the same person, who holds an International Society for the Advancement of Kinanthropometry (ISAK) qualification (Level 2).

#### Countermovement jump test.

The CMJ height was assessed three times using an optical detection system comprised of a transmitting and receiving bar with associated software (*Optojump, Microgate, Bolzano, Italy*), through the flight time method with a 45-second rest between repetitions, and the best score was recorded. Subjects were instructed to keep their hands on their hips throughout all jumps, and the depth of each jump was self-selected. Furthermore, they were instructed to jump as high as possible and keep their limbs extended during the flight phase.

#### Unilateral horizontal jump test.

The unilateral horizontal jump with left (HJL) and right (HJR) legs performance was assessed using a measuring tape (from the line to the heel) through three attempts, with a 45-second rest between jumps, and the best result was used for further analysis. Leg swing was permitted during propulsion, and one-leg landings were mandatory with hands on hips. Only jumps where participants-maintained balance and held the final position for at least two seconds were recorded.

#### Triple unilateral horizontal jump test.

The triple unilateral horizontal jump with left (3HJL) and right (3HJR) legs performance was assessed using a measuring tape (from the line to the heel) through three attempts, with a 60-second rest between jumps, and the best result was used for further analysis. Subjects were instructed to take three maximal hops forward (landing on the same leg throughout) to minimize ground contact times after the first and second hops. Leg swing was permitted during propulsion, and one-leg landings were mandatory with hands on hips. Only jumps where participants-maintained balance and held the final position for at least two seconds were recorded.

#### Linear running sprint test.

Running speed was evaluated by 25-m sprint times with split times at 5-m, 10-m, and 25-m. Time was recorded with photoelectric cells (*Witty, Microgate, Bolzano, Italy*). The front foot was placed 0.5 m before the first timing gate whilst adopting a 2-point staggered stance. Timing gates were placed at 0.75 m height and 1.5 m distance between each other. The 25-m sprint was performed two times, separated by at least 3 min of passive recovery. The best time of each split was used for statistical analysis.

#### 180° change of direction test.

A 10-m shuttle-sprint test was performed. The subject sprinted from the start/finish line, crossed the 5-m line with the either right or left foot, and turned 180° to sprint back to the start/finish line. The front foot was placed 0.5 m before the first timing gate whilst adopting a 2-point staggered stance. Timing gates were placed at 0.75 m height and 1.5 m distance between each other (*Witty, Microgate, Bolzano, Italy*). Players executed two valid trials with each foot (first, left, and second, right) in alternating order, separated by two minutes, with the fastest retained for calculations.

#### V-cut test.

In the V-cut test, players performed a 25-m sprint with 4 CODs of 45º each 5 m apart [26]. The front foot was placed 0.5 m before the first timing gate whilst adopting a 2-point staggered stance. Timing gates were placed at 0.75 m height and 1.5 m distance between each other (*Witty, Microgate, Bolzano, Italy*). For the trial to be valid, players had to pass the line, drawing on the floor between each pair of cones, with one foot completely at every turn. If the trial was considered as failed, a new trial was allowed. Players executed two valid trials. The distance between each pair of cones was 0.7 m. The time of the fastest trial was retained.

### Statistical analyses

Data are presented as mean ±  SD (standard deviation). To prove the normality of data distribution and the homogeneity of variances, the Kolmogorov-Smirnov and Levene tests were conducted. All variables were normally distributed within each group. Consequently, parametric statistical tests were used. To examine the reliability, pairwise comparisons were first applied. Between-session reliability analysis was computed using: i) a 2-way random intraclass correlation coefficient (ICC) with an absolute agreement and 95% confidence intervals, and ii) the coefficient of variation (CV). The ICC was interpreted as poor (<0.5), moderate (0.5–0.74), good (0.75–0.9), or excellent (>0.9) [27]. Coefficients of variation were considered acceptable if < 10% [28]. A repeated measures ANOVA was performed to analyze the within-group differences between three times (pre-test, post-test, and detraining). A Bonferroni’s post-hoc correction was used to determine pairwise comparisons. A one-way ANOVA was used to determine between-group (pre-PHV vs. mid-PHV vs. post-PHV) differences at each time. If significance appears, Bonferroni’s post-hoc correction was performed for pairwise comparisons. Furthermore, all significant differences at either post-test or detraining were double-checked, when significant differences between groups were at pre-test, using an ANCOVA using the pre-test values as a co-variable to determine the between-group comparisons. A post-hoc comparison (Bonferroni correction) was used to examine mean differences among groups (pre-PHV vs. mid-PHV vs. post-PHV). A significant level was set at *p* <  0.05. Hedges’ g effect sizes were calculated for within-group comparisons due to the small sample size. The following criteria were adopted for interpreting the magnitude of effect size (g) between test measures: Trivial g <  0.2; Small 0.2 ≥ g <  0.5, Medium 0.5 ≥  g <  0.8, Large g ≥  0.8. All statistical analyses were performed using SPSS (*version 25, IBM, New York, NY, USA)* and Microsoft Excel (*version 2016, Microsoft Corp., Redmond, WA, USA*).

## Results

All ICC values were good (ICC >  0.75), except for 5- and 10-meters time. All CV values were acceptable (1.87% to 4.0%). Reliability results are shown in Table 3 displaying high and acceptable relative and absolute reliability. Tables 4 and 5 show within-group differences after both training and detraining periods. Significant improvements (p <  0.05, ES =  0.10 to 1.16) for pre-PHV were found in age, height, body mass, HJL, HJR, 3HJL, and 3HJR between pre- and post-tests. A significantly (p <  0.05, ES =  0.20 to 1.51) greater result was also found between pre- and detraining in age, body mass, 3HJL, 3HJR, and 180° CODR.

**Table 3:** Reliability data for performance measurements in the pool sample (n =  35).

	ICC (CI95%)	CV (CI95%)
**CMJ (cm)**	0.94 (0.80; 0.99)	1.87 (1.46; 2.63)
**CMJL (cm)**	0.98 (0.97; 0.99)	4.0 (3.14; 5.67)
**CMJR (cm)**	0.97 (0.95; 0.99)	3.77 (2.95; 5.32)
**HJL (cm)**	0.86 (0.76; 0.95)	1.87 (1.46; 2.63)
**HJR (cm)**	0.92 (0.83; 0.96)	1.12 (0.88; 1.57)
**3HJL (cm)**	0.85 (0.68; 0.91)	2.65 (2.23; 3.59)
**3HJR (cm)**	0.88 (0.76; 0.95)	2.59 (2.18; 3.49)
**5-m (s)**	0.73 (0.55; 0.84)	2.91 (2.44; 3.72)
**10-m (s)**	0.73 (0.55; 0.84)	2.89 (2.37; 3.65)
**20-m (s)**	0.84 (0.71; 0.91)	1.82 (1.55; 2.31)
**25-m (s)**	0.88 (0.77; 0.93)	1.61 (1.33; 2.07)
**180º CODL (s)**	0.93 (0.85; 0.97)	2.19 (1.71; 3.08)
**180º CODR (s)**	0.93 (0.85; 0.97)	2.11 (1.65; 2.96)
**V-cut (s)**	0.95 (0.89; 0.98)	1.58 (1.24; 2.22)

Note: CMJ: bilateral countermovement jump; CMJL and CMJR: unilateral countermovement jumps with left and right legs; HJL and HJR: unilateral horizontal jumps with left and right; 3HJL and 3HJR: triple unilateral horizontal jumps with left and right; 180º CODL and 180º CODR: a shuttle-sprint (5 +  5 m) with one change of direction (COD) of 180º with left and right; V-cut: a 25-m sprint test with 4 COD of 45º each 5 m.

**Table 4:** Training and detraining effects (mean ±  SD) for anthropometric and performance variables for the three-maturity groups based on the peak height velocity (PHV).

	Pre-PHV (n = 9)			Mid-PHV (n = 17)			Post-PHV (n = 9)		
	Pre-	Post-	Detraining	Pre-	Post-	Detraining	Pre-	Post-	Detraining
**Age (y)**	12.1 ± 0.58	12.3 ± 0.58^*^	12.4 ± 0.58^*^^	13.4 ± 0.66	13.6 ± 0.65^*^	13.7 ± 0.65^*^^	14.4 ± 0.35	14.6 ± 0.36^*^	14.7 ± 0.36^*^^
**Height (cm)**	155.4 ± 7.9	156.3 ± 8.6^*^	156.5 ± 8.2	174.6 ± 7.2	175.7 ± 7.1^*^	177.1 ± 7.2^*^^	183.2 ± 8.4	184.3 ± 8.4^*^	184.6 ± 8.0
**Body mass (kg)**	43.8 ± 9.0	45.8 ± 9.3^*^	45.6 ± 9.2^*^	57.6 ± 9.3	59.4 ± 9.1^*^	60.3 ± 9.2^*^^	65.4 ± 6.9	67.4 ± 7.0^*^	68.0 ± 7.0
**APHV (y)**	-1.51 ± 0.62	-1.28 ± 0.71	-1.26 ± 0.63	0.11 ± 0.45	0.26 ± 0.51	0.27 ± 0.51^*^	1.31 ± 0.42	1.33 ± 0.87	1.47 ± 0.39
**CMJ (cm)**	24.9 ± 4.13	25.7 ± 4.44	25.5 ± 3.15	27.5 ± 4.0	28.2 ± 3.81	28.5 ± 3.99	28.9 ± 7.3	30.6 ± 6.98^*^	31.3 ± 6.92^*^
**CMJL (cm)**	12.7 ± 2.25	15.1 ± 1.86	14.6 ± 2.25	14.3 ± 3.44	15.9 ± 3.35	16.6 ± 2.92^*^	13.8 ± 5.60	16.7 ± 5.06^*^	16.5 ± 4.81^*^
**CMJR (cm)**	11.8 ± 2.51	13.3 ± 2.25	13.6 ± 1.27	12.8 ± 2.72	14.6 ± 3.35^*^	15.6 ± 2.90^*^	14.6 ± 5.03	16.9 ± 4.63^*^	17.2 ± 4.99^*^
**HJL (cm)**	123.9 ± 13.0	132.1 ± 11.2^*^	131.1 ± 14.1	140.6 ± 19.3	155.1 ± 18.2^*^	157.1 ± 15.8^*^	141.8 ± 23.6	154.3 ± 27.3^*^	154.2 ± 29.5^*^
**HJR (cm)**	119 ± 14.4	132.9 ± 11.6^*^	127 ± 12.0	137.5 ± 15.1	154 ± 14.9^*^	149.5 ± 18.4^*^	143.3 ± 26.9	154.7 ± 27.1^*^	155.9 ± 27.4^*^
**3HJL (cm)**	387.7 ± 45.3	445.3 ± 54.9^*^	441.6 ± 51.7^*^	463 ± 49.8	502.2 ± 58.4^*^	494.9 ± 54.6^*^	463.7 ± 79.2	491.9 ± 85.2	507.2 ± 94.1^*^
**3HJR (cm)**	395.1 ± 32.2	440.2 ± 45.7^*^	423.7 ± 35.8^*^	442.2 ± 48.6	491.9 ± 65.7^*^	476.5 ± 51.8^*^	492.1 ± 72.5	491 ± 84.8	503.6 ± 88.2
**5-m (s)**	1.15 ± 0.06	1.16 ± 0.07	1.12 ± 0.06^	1.16 ± 0.09	1.17 ± 0.07	1.16 ± 0.08	1.15 ± 0.11	1.13 ± 0.10	1.13 ± 0.09
**10-m (s)**	2.00 ± 0.09	1.99 ± 0.08	1.98 ± 0.08	1.99 ± 0.13	2.00 ± 0.11	1.99 ± 0.13	1.96 ± 0.15	1.95 ± 0.15	1.93 ± 0.16
**20-m (s)**	3.52 ± 0.16	3.51 ± 0.15	3.48 ± 0.16	3.49 ± 0.24	3.47 ± 0.20	3.47 ± 0.24	3.41 ± 0.28	3.38 ± 0.29	3.36 ± 0.27
**25-m (s)**	4.29 ± 0.19	4.25 ± 0.18	4.24 ± 0.18	4.21 ± 0.33	4.20 ± 0.25	4.18 ± 0.29	4.12 ± 0.37	4.08 ± 0.36	4.05 ± 0.36
**180º CODL (s)**	2.93 ± 0.13	2.90 ± 0.11	2.87 ± 0.09	2.92 ± 0.19	2.83 ± 0.14^*^	2.81 ± 0.13^*^	2.88 ± 0.20	2.82 ± 0.21	2.79 ± 0.19
**180º CODR (s)**	2.95 ± 0.13	2.87 ± 0.10	2.78 ± 0.09^*^	2.93 ± 0.19	2.86 ± 0.19	2.80 ± 0.12^*^	2.91 ± 0.20	2.88 ± 0.25	2.79 ± 0.22^*^
**V-cut (s)**	7.69 ± 0.29	7.50 ± 0.35	7.50 ± 0.25	7.61 ± 0.54	7.38 ± 0.47^*^	7.30 ± 0.34^*^	7.40 ± 0.44	7.24 ± 0.52	7.13 ± 0.52^*^

*Note.* APHV: age at peak height velocity; CMJ: bilateral countermovement jump; CMJL and CMJR: unilateral countermovement jump with left and right legs; HJL and HJR: unilateral horizontal jump with left and right; 3HJL and 3HJR: triple unilateral horizontal jump with left and right; 180º CODL and 180º CODR: a shuttle-sprint (5 +  5 m) with one change of direction (COD) of 180º with left and right; V-cut: a 25-m sprint test with 4 COD of 45º each 5 m.

* p <  0.05 vs. pre-test; ^p <  0.05 vs. post-test.

**Table 5:** Training and detraining effect size (ES) for anthropometric and performance variables for the three-maturity groups based on the peak height velocity (PHV).

	ES (Pre-PHV)			ES (Mid-PHV)			ES (Post-PHV)		
	Pre- vs Post-	Pre- vs. Det	Post- vs. Det	Pre- vs Post-	Pre- vs. Det	Post- vs. Det	Pre- vs Post-	Pre- vs. Det	Post- vs. Det
**Age (y)**	**0.31**	**0.53**	**0.22**	**0.26**	**0.45**	**0.20**	**0.53**	**0.89**	**0.35**
**Height (cm)**	**0.10**	0.14	0.03	**0.15**	**0.34**	**0.19**	**0.13**	0.17	0.04
**Body mass (kg)**	**0.22**	**0.20**	-0.02	**0.20**	**0.29**	**0.09**	**0.30**	0.38	0.08
**APHV (y)**	0.35	0.19	0.13	0.29	**0.33**	0.03	0.04	0.42	0.23
**CMJ (cm)**	0.21	0.19	-0.06	0.18	0.24	0.07	**0.23**	**0.34**	0.11
**CMJL (cm)**	1.15	0.86	-0.22	0.48	**0.73**	0.22	**0.55**	**0.52**	-0.05
**CMJR (cm)**	0.62	0.95	0.18	**0.61**	**1.01**	0.31	**0.46**	**0.51**	0.07
**HJL (cm)**	**0.68**	0.53	-0.08	**0.77**	**0.94**	0.12	**0.49**	**0.47**	0.00
**HJR (cm)**	**1.07**	0.60	-0.50	**1.10**	**0.72**	-0.27	**0.42**	**0.46**	0.00
**3HJL (cm)**	**1.15**	**1.11**	-0.07	**0.72**	**0.61**	-0.13	0.34	**0.50**	0.17
**3HJR (cm)**	**1.16**	**0.84**	-0.41	**0.87**	**0.69**	-0.26	-0.01	0.14	0.15
**5-m (s)**	-0.19	0.52	**0.69**	-0.09	0.05	0.15	0.20	0.21	0.01
**10-m (s)**	0.04	0.27	0.26	-0.01	0.03	0.04	0.05	0.16	0.11
**20-m (s)**	0.08	0.23	0.15	0.04	0.10	0.07	0.12	0.18	0.05
**25-m (s)**	0.22	0.27	0.05	0.05	0.11	0.07	0.11	0.18	0.08
**180º CODL (s)**	0.21	0.57	0.37	**0.54**	**0.69**	0.18	0.34	0.49	0.13
**180º CODR (s)**	0.71	**1.51**	0.85	0.35	**0.80**	0.39	0.11	**0.56**	0.40
**V-cut (s)**	0.57	0.68	0.00	**0.47**	**0.71**	0.18	0.32	**0.56**	0.22

*Note.* APHV: age at peak height velocity; CMJ: bilateral countermovement jump; CMJL and CMJR: unilateral countermovement jump with left and right legs; HJL and HJR: unilateral horizontal jump with left and right; 3HJL and 3HJR: triple unilateral horizontal jump with left and right; 180º CODL and 180º CODR: a shuttle sprint (5 +  5 m) with one change of direction (COD) of 180º with left and right; V-cut: a 25-m sprint test with 4 COD of 45º each 5 m. * p <  0.05 vs. pre-test; ^p <  0.05 vs. post-test. Bold values were considered as significant differences (p < 0.05).

Significant improvements (p <  0.05, ES =  0.15 to 1.10) for mid-PHV were found in age, height, body mass, CMJR, HJL, HJR, 3HJL, 3HJR, 180° CODL, and V-cut test between pre- and post-tests. A significantly (p <  0.05, ES =  0.29 to 1.01) greater result was also found between pre- and detraining in age, height, body mass, APHV, CMJR, HJL, HJR, 3HJL, 3HJR, 180°CODL, 180°CODR, and V-cut test. Furthermore, age, height, and body mass were significantly (p <  0.05, ES =  0.09 to 0.20) greater from post-test to detraining.

Significant improvements (p <  0.05, ES =  0.13 to 0.55) for mid-PHV were found in age, height, body mass, CMJ, CMJR, HJL, and HJR between pre- and post-tests. A significantly (p <  0.05, ES =  0.34 to 0.89) greater result was also found between pre- and de-training in age, height, CMJ, CMJR, HJL, HJR, 3HJL, 180°CODR, and V-cut test.

At the pre-test, the post-PHV group showed higher values in HJR, 3HJL, and 3HJR in comparison to pre-PHV and mid-PHV (p <  0.05). At post-test, significant differences (p <  0.05) were established between pre-PHV and mid-PHV and post-PHV in HJL and HJR. However, when ANCOVA was applied (i.e., HJR), no significant differences (p >  0.05) were found between any group. At detraining, CMJ was significantly (p <  0.05) greater in post-PHV compared to pre-PHV. Furthermore, mid-PHV (p =  0.01) and post-PHV (p =  0.048) showed a significantly better HJL than pre-PHV. Finally, despite finding significant differences in HJR (pre-PHV vs. mid-PHV and pre-PHV vs. post-PHV) and 3HJR (pre-PHV vs. post-PHV), significant differences disappeared when the pre-test was controlled for.

## Discussion

The main aims of the current study were: 1) to examine the effect of 8 weeks’ strength training on athletic performance in youth basketball athletes and establish its response in different maturity status groups, and 2) to investigate the impact of strength training cessation whilst maintaining basketball training over eight weeks on jumping, linear sprinting and COD speed in young highly trained basketball players. The main findings were that the combined (i.e., vertical and horizontal) strength training regimen had favorable outcomes for most unilateral jumps, particularly those with a horizontally oriented force-vector, independent of maturity status. Moreover, the subsequent detraining period yielded positive effects on certain high-intensity actions such as COD ability. Specifically, mid-PHV and post-PHV players showed a significant single and multiple COD improvement in the detraining period.

Horizontal jumps require greater hamstring activity (i.e., semimembranosus and biceps femoris muscles) compared to vertical jumps and an opposite activity of the rectus femoris, that is, horizontal jumps seem to be more hip dominant while vertical are knee dominant [29]. Moreover, unilateral horizontal jumps include higher demands at the kinetic level (i.e., peak force) compared to unilateral vertical and lateral jumps [30]. Based on present findings, including an improvement of horizontal, unilateral jumps irrespective of maturity status, it is expected that the present training protocol can potentially influence muscle activity of muscle groups critically involved in key muscle actions during horizontal jumping performance (i.e., knee and hip extensors). The present protocol incorporates knee-dominant exercises (e.g., lunge and squat variations), hip-dominant exercises (e.g., hip thrust, hip extension, single-leg deadlift), and sprinting. Based on data from previous studies, it is suggested that these exercises may lead to higher peak activity of the posterior chain musculature, potentially enhancing unilateral horizontal jumping performance in youth athletes. However, many of these resistance exercises were performed with higher repetitions, excluding the last two weeks, deviating from what is considered a reference for power training. This may help explain the differences found in other studies that include a similar population (post-PHV basketball players) [31]. Current improvements were lower than a previous 6-week RPA training study, including the leg-press exercise (one or two blocks of 5 sets ×  5 repetitions with 20-sec passive recovery between sets and three between blocks, twice/week) [31]. Along the same line, a 10-week training program including squats with the load that maximized propulsive power output (two blocks of 5 sets ×  5 repetitions interspersed with variable passive recovery between sets and three min between blocks) allowed higher improvements in post-PHV basketball players [32]. Both studies included exercises with load distinctly linked to the neuromuscular potential of the athlete [33] in a horizontal force-vector. This load-maximizing propulsive power output has been associated with increased recruitment of fast-twitch muscle fibers, typically associated with the execution of high-intensity actions [34]. After getting involved in this training approach, subjects are more likely to improve horizontal-oriented activities, such as horizontal jumping.

Detraining is linked to a decline in performance and the dissipation of previously acquired physiological adaptations that occur after the cessation of training [35]. In the present study, a long-term detraining period ( > 4 weeks) was employed, anticipating a decline in neuromuscular characteristics, particularly in highly trained athletes [35]. These characteristics include reductions in muscle mass, EMG activity, and strength/power expression after detraining. Based on these results, it would be expected that participants should experience a decrease in their performance in most high-intensity actions, especially in jumping activities. However, despite the removal of strength training, we found a positive effect of detraining in post-PHV and mid-PHV athletes in some unilateral vertical jumping tasks compared to pre-PHV, and general maintenance of other physical qualities. These results exhibit similarities with one of the few studies that analyzed a long-term detraining period (8 weeks) after a strength training program in school sports academy subjects with different maturational statuses [22]. In that study, post-PHV subjects showed a slight improvement in horizontal jump after the training cessation period [22]. In the present study, it is necessary to consider that the athletes continued to participate in basketball, a sport inherently plyometric in nature and involving activities analogous to those in the testing battery (i.e., jumping, sprinting, directional changes). This ongoing engagement in basketball activity likely provided a stimulus contributing to the maintenance of their performance. Taken together, these pieces of evidence seem to suggest the possibility of biological maturation mediating the effects of training on physical performance, particularly in unilateral vertical jumping.

The enhanced performance for some tasks could be associated with alterations in the neuromuscular system during growth and maturation (e.g., tendon stiffness, motor unit recruitment, etc.), especially in adolescence, resulting in an improved stretch-shortening cycle function [36]. Thus, it is possible that subjects experience a considerable improvement in their vertical jump performance at the moment of PHV but also 6, 12, and 18 months after PHV [37]. It also suggests that the greater natural development of jumping ability could function as a confounding variable in the adaptation to the detraining period, preventing the decay of unilateral vertical jumping performance among pubertal and post-pubertal subjects. Another hypothesis is that detraining may enable recovery from any signals of overtraining, resulting in improved performance via reductions in exercise volume to permit super-compensation. In the present study, subjects who experienced 12 hours of combined basketball and played two competitive matches per week may become more likely to overtrain. Elite youth basketball players often experience intense and prolonged training periods and engage in early sport specialization [38]. Consequently, in terms of injury risk stratification, they could be considered as “High-risk athletes” or “Load-sensitive,” assuming that it is necessary to adapt their training opportunities, notably by reducing the training load [39]. Thus, introducing training periods where athletes obtain adaptations solely through sport-specific practice, contributing to a reduction in training load, may enhance energy levels and induce variation in training, a key aspect of effective training and periodization [40]. In contrast, previous studies conducted on youth basketball players, evaluating the effect of detraining after a combined training program, demonstrated that after eight weeks of detraining (while maintaining basketball practice), athletes exhibited a performance decline in most jump tests [41,42]. This divergence in results raises the hypothesis that the training demands may mediate the adaptation to detraining. Considering the performance level of the sample in the current study, it may have experienced higher physical, physiological, and technical demands, shaping the observed outcomes.

Interestingly, the observed effects indicate that the current training protocol benefits unilateral vertical jumping in post-PHV subjects, especially with the right leg. Moreover, these findings are superior to those reported in other studies with post-PHV basketball players [16,32]. In those studies, isolated methods were applied (e.g., repeated-power ability and eccentric overload training), and exercises were conducted in only one force vector (e.g., horizontal, or vertical). Indeed, combined training across different regions of the force-velocity continuum involving diverse force vectors appears to be most beneficial for improving vertical jump performance in post-PHV subjects [13], explaining between studies discrepancies. In the triple hop distance, it was possible to observe a positive effect of detraining and the training program among pre-PHV and mid-PHV subjects. The triple hop distance is extensively used to assess rebound jumping ability in a horizontal direction [43]. Horizontal jumps demand a practical ability to transfer linear momentum directly from the ground to propel the body’s center of mass horizontally. Compared to the single hop test, a greater capacity is required to control and coordinate the movement during the triple hop test. Thus, the current protocol may have influenced dynamic balance in athletes and proprioception in and around PHV, aligning with sensitive periods for balance stimulation that encompass pre-puberty [44]. However, it is also emphasized that balance stimulation is required during puberty [44].

The vertical CMJ is a widely recognized test for evaluating the jumping ability of team sports players, with a particular emphasis on basketball players. Following the present findings, no positive effect of the protocol on CMJ was observed, except for post-PHV players. Nevertheless, when compared to a similar sample, the identified results exhibited a lesser magnitude [32]. These disparities underscore the necessity of axial loading, particularly when lifting submaximal loads, to elicit positive adaptations at the level of CMJ. This hypothesis is reinforced by the outcomes of a prior study involving eccentric overload training with movements in horizontal and lateral directions, i.e., reduced axial load, which yielded trivial to small CMJ improvements [16]. However, the results of detraining in the post-PHV subjects were higher than those found in previous studies, including plyometric and complex training and an 8-week detraining period [41,42]. Indeed, the CMJ is a widely utilized tool for neuromuscular assessment, encompassing considerations of super-compensation and fatigue among athletes [45]. Considering its sensitivity to detect fatigue, the detraining period may have alleviated residual fatigue and generated positive changes in kinetic and kinematic parameters, resulting in improved outcomes.

The current training program significantly improved during the COD tests, including less and more aggressive cutting angles among Mid-PHV subjects. The 180° change of direction test involves high braking requirements [46], and better performance is associated with higher propulsive and braking forces (mainly horizontal) in the final foot contact [47]. Athletes who possess higher eccentric and isometric strength are also reported to display superior COD performance [48]. On the other hand, faster performance during a 45º change of direction includes lower contact times, lower time spent braking, lower braking impulse, and higher propulsive impulse are required for a faster performance during a 45°change of direction [48]. Following the training program, participants exhibited faster times when performing change-of-direction movements, irrespective of the angle. The enhancements in COD ability following combined training could be attributed to potential mechanisms involving neuromuscular adaptations, including improved motor unit recruitment, firing frequencies, muscle force, and rate of force development in the lower limbs, also observed through maturation [36]. In this regard, higher improvements in COD speed were also observed at the moment of PHV [37], suggesting that combining force production improvement methods and its natural development may underlie improvements in COD ability.

According to scientific evidence, the enhancement of speed over short distances ( ≤ 20 meters) has been advocated through the application of free and resisted sprinting protocols [49]. Despite the current protocol incorporating free sprint and resisted situations (up to 10% of body mass), no significant effects were observed across different distances, regardless of maturity status. Nevertheless, results from other studies, which encompass distinct training regimens (e.g., repeated sprinting, repeated power ability, plyometrics, strength, change-of-direction, combined, small-sided games, and high-intensity interval training) exhibit variations in magnitude, even when including a similar sample (i.e., youth basketball players) [14,32,50–52]. However, the samples differ in performance level, as the present sample includes individuals who compete at national and even international levels. In contrast, in other studies, most participants exhibit a lower performance level (i.e., regional or school level). Consequently, individuals with lower performance levels, characterized by lower baseline fitness and skill levels, are more likely to experience rapid improvements with relatively simple training stimuli. Moreover, it is noteworthy that our study incorporated a comparatively lower volume of sprinting (480 meters) when contrasted with the recommendations to maximize speed development throughout childhood and adolescence [53]. Interestingly, despite the athletes having increased their body weight by ~ 2 kg (Table 4), their sprint performance values remained constant. This suggests an increase in momentum, which can be considered a positive adaptation to the training programme whereby the heavier the athlete, the more net force is required to accelerate their body mass.

It is essential to acknowledge some limitations in the present study. Specifically, our results are only sometimes applicable to other team sports due to the unique characteristics of basketball, such as their anthropometry and court size. Second, this study only examined the effects of the same strength training and its cessation on different maturity status groups. Future studies should examine the effects of combined strength training and several force vectors on different maturity status groups. Furthermore, the impact of strength training based on the maturation status over a short-term period (i.e., four weeks) should also be assessed.

## Conclusion

The combined training had favorable outcomes for most unilateral jumps, particularly those with a horizontally oriented force-vector, independent of maturity status. Moreover, the subsequent detraining period positively affected specific high-intensity actions, albeit variations were noted across different maturation stages.

### Practical applications

The current study adds practical value to young basketball players’ development. In this regard, the current strength training may mainly enhance those horizontal-oriented activities with an exponential improvement of COD ability after the strength training cessation. Thus, as COD ability is one of the critical factors in basketball performance, it can help develop offensive and defensive situations better. Finally, national, and international basketball Championships are commonly developed during ten to fifteen days during a competitive season. The main aim is increased preparedness to reach such a time point in the best physical fitness. As such, the current findings can help practitioners design more effective resistance-based training programs to enhance athletic performance to resultantly increase the chances of winning the Championship in male youth basketballers.

## Supporting information

S1 Data Data plos one V2. (XLSX)
